# Advances in Preclinical Research Models of Radiation-Induced Cardiac Toxicity

**DOI:** 10.3390/cancers12020415

**Published:** 2020-02-11

**Authors:** Rachel A. Schlaak, Gopika SenthilKumar, Marjan Boerma, Carmen Bergom

**Affiliations:** 1 Department of Pharmacology & Toxicology, Medical College of Wisconsin, Milwaukee, WI 53226, USA; rachelmeyer@mcw.edu; 2 Medical Scientist Training Program, Medical College of Wisconsin; Milwaukee, WI 53226, USA; gsenthilkuma@mcw.edu; 3 Department of Medicine, Medical College of Wisconsin, Milwaukee, WI 53226, USA; 4 Division of Radiation Health, Department of Pharmaceutical Sciences, The University of Arkansas for Medical Sciences, Little Rock, AR 72205, USA; MBoerma@uams.edu; 5 Department of Radiation Oncology, Medical College of Wisconsin, Milwaukee, WI 53226, USA; 6 Cardiovascular Center, Medical College of Wisconsin, Milwaukee, WI 53226, USA; 7 Cancer Center, Medical College of Wisconsin, Milwaukee, WI 53226, USA

**Keywords:** radiation biology, thoracic radiation therapy, normal tissue toxicity, cardiopulmonary toxicity, small animal irradiators, image-guided radiotherapy, cardiotoxicity, radiation-induced heart disease

## Abstract

Radiation therapy (RT) is an important component of cancer therapy, with >50% of cancer patients receiving RT. As the number of cancer survivors increases, the short- and long-term side effects of cancer therapy are of growing concern. Side effects of RT for thoracic tumors, notably cardiac and pulmonary toxicities, can cause morbidity and mortality in long-term cancer survivors. An understanding of the biological pathways and mechanisms involved in normal tissue toxicity from RT will improve future cancer treatments by reducing the risk of long-term side effects. Many of these mechanistic studies are performed in animal models of radiation exposure. In this area of research, the use of small animal image-guided RT with treatment planning systems that allow more accurate dose determination has the potential to revolutionize knowledge of clinically relevant tumor and normal tissue radiobiology. However, there are still a number of challenges to overcome to optimize such radiation delivery, including dose verification and calibration, determination of doses received by adjacent normal tissues that can affect outcomes, and motion management and identifying variation in doses due to animal heterogeneity. In addition, recent studies have begun to determine how animal strain and sex affect normal tissue radiation injuries. This review article discusses the known and potential benefits and caveats of newer technologies and methods used for small animal radiation delivery, as well as how the choice of animal models, including variables such as species, strain, and age, can alter the severity of cardiac radiation toxicities and impact their clinical relevance.

## 1. Introduction

Over 17 million cases of cancer were diagnosed worldwide in 2018, and roughly 9.5 million cancer deaths were reported [1]. Since the early 1900s, ionizing radiation has been used to treat cancers [2], and today radiation remains a major modality in cancer treatment, with over half of all cancer patients receiving radiation therapy (RT). Because of overall growth and aging of the population, it is estimated that by 2040 the global incidence of cancer will rise to over 27 million new cases, and more than 16 million cancer deaths will occur [1]. As a consequence, the global cancer burden will give rise to a growing population of survivors that may develop short- and long-term side effects of cancer therapy.

**Figure 1:**
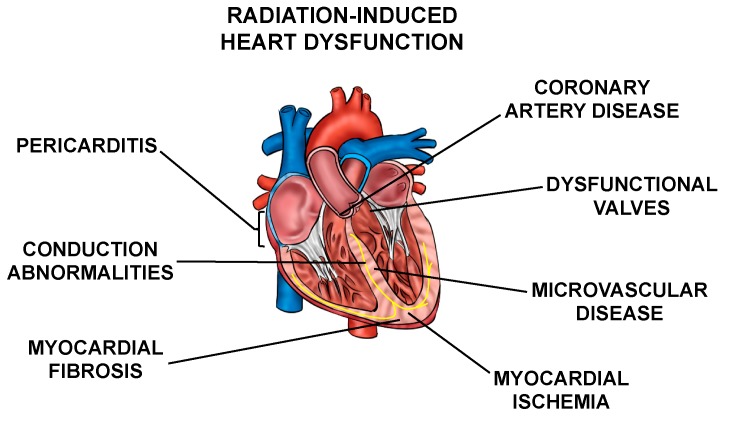
Cardiac radiation exposure causes a number of abnormalities. Exposure of the heart and surrounding vasculature to radiation may lead to several adverse structural and functional changes in the heart, in this article collectively referred to as radiation-induced heart dysfunction.

Normal tissue toxicities, mainly in the heart and lungs, can occur after RT in patients with thoracic tumors. The most common toxicities include acute pneumonitis and chronic fibrosis due to radiation exposure of the lung [3,4], and cardiac dysfunction, including pericarditis, ischemic heart disease, conduction abnormalities, myocardial fibrosis, and valvular abnormalities collectively called radiation-induced heart dysfunction (RIHD) (Figure 1) from incidental radiation to the heart and surrounding vasculature [5,6,7,8,9,10]. These side effects may present clinically months to years after RT, affecting patient quality of life and at times even leading to increased mortality [6,9,10,11,12,13,14,15,16]. For example, patients who received tangential RT for left-sided breast cancer in the 1970s and 1980s had an increased risk for cardiovascular mortality at 15 years post-treatment [17]. In patients that received mediastinal radiation for Hodgkin’s disease in the 1960s–1990s, there was a higher prevalence of cardiac abnormalities when compared to the Framingham population [18]. In addition, non-small cell lung cancer (NSCLC) patients may experience RIHD within two years of radiation exposure [14,19,20,21,22]. Numerous other groups have highlighted similar increases in cardiac toxicity-related morbidity and mortality among patients that have received thoracic radiation [23,24,25,26,27].

A number of advances in radiation oncology have made radiation delivery more precise and allow more effectively delivery of doses to the target volume while reducing the radiation doses to surrounding normal tissues [28,29,30,31,32,33]. However, numerous studies have shown that modern RT technology has not fully eliminated the risk of RIHD [34,35,36]. In breast cancer patients, it has been estimated that there is an approximately 4–16% relative increase in heart disease and/or major coronary events for each 1 Gy in mean heart dose received [6,9,10]. A recent national multicenter NSCLC trial and other single institution reviews have shown a correlation between early death and radiation dose to the heart [12,14,19,20,21,22]. However, efforts towards severely limiting incidental heart dose could potentially compromise RT’s effectiveness in treating tumors in patients with mediastinal lymphomas, thymomas, and breast, lung, or esophageal cancers [7]. 

Thus, there is a need for understanding the mechanisms by which radiation causes cardiovascular disease, and potentially providing targeted interventions that prevent or reverse RIHD while maintaining optimal radiation doses to the target(s) for maximum tumor control. There are several preclinical studies that aim to elucidate the molecular and cellular mechanisms of RIHD (reviewed in [37,38]). However, translating the biological mechanisms involved in the normal tissue radiation response into therapeutic targets in patients remains a critical challenge given the current limitations of preclinical models in accurately characterizing all facets of human disease. Developing preclinical models of RT with improved representation of human physiology, as well as appropriate modeling of current radiation delivery in the clinic will contribute to overcoming this challenge.

## 2. Small Animal Radiotherapy Delivery

### 2.1. Target Volume and Methods of Radiation Delivery in Preclinical Studies of RIHD

**Figure 2:**
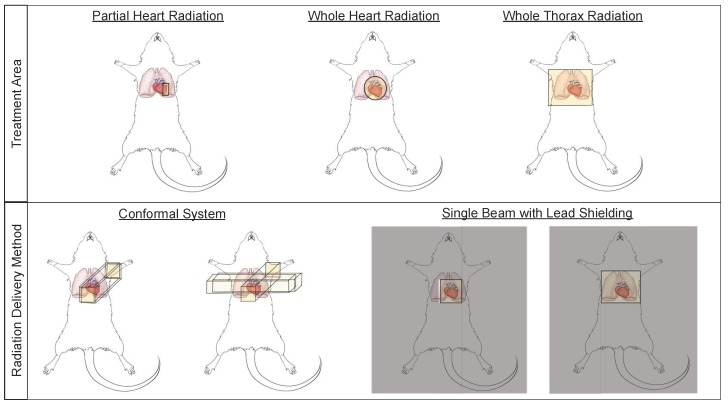
Small animal irradiation techniques allow a wide variety of cardiac and pulmonary exposures and a number of delivery methods. Schematic illustrations of radiation field options that are commonly used to deliver cardiac radiation (top panel), and methods of radiation delivery (bottom panel) in small animal models of radiation-induced heart dysfunction (RIHD).

Small animal models have been used to study cardiac radiation toxicity for many decades [39,40,41] based on the physiological similarities of these models to humans [42]. In these models, many current treatment paradigms exist to study RIHD, including whole thorax, whole heart, and partial heart irradiation (Figure 2). Whole thorax irradiation is a method used extensively in the past [43,44,45,46], as it does not require precise image guidance during radiation delivery. Unfortunately, when irradiating the whole thorax, damage occurs not only to the heart, but also the lungs, a dose limiting organ, thereby increasing morbidity and mortality and making it difficult to distinguish whether the resulting damage is from heart or lung irradiation alone, or due to irradiation of both the heart and lungs [45,47] (see also Section 2.3). Modern thoracic RT techniques aim to shield the heart and other organs at risk. However, more advanced techniques and modalities such as intensity-modulated radiation therapy (IMRT) and proton therapy have not been extensively explored in detail in pre-clinical normal tissue toxicity models, although modern small animal irradiators can allow for arc therapy and this technique is beginning to be utilized in pre-clinical models [48].

Whole heart irradiation has been a relatively new technique to deliver radiation in preclinical models. This technique requires imaging, such as fluoroscopy, x-ray or cone beam computed tomography (CBCT), to accurately deliver radiation to the heart using beam sizes that are mainly focused on the heart, thus limiting lung doses [49,50,51,52]. The use of image-guidance aims to irradiate smaller target volumes with improved accuracy. Currently, two systems to perform CBCT-guided image-guided local irradiation of small experimental animals are commercially available, which can provide accuracy that is similar to clinical RT: the Small Animal Radiotherapy Research Platform (SARRP, Xstrahl) and the X-Rad SmART research platform (Precision X-ray) [53]. In addition, several other non-commercial systems have been developed to deliver localized, image-guided radiotherapy [51,54,55,56,57,58,59]. These image guided systems are conformal systems (i.e., have multiple beam angles), which allow the systems to deliver high doses to small, targeted regions [54,60] and aim to mimic clinical RT devices (Figure 2).

Research groups that do not have access to image-guided radiation delivery platforms rely on lead shielding to target the heart while aiming to reduce the radiation dose to surrounding normal tissues (Figure 2) [61,62]. In addition to decreased accuracy of targeting the heart and non-uniform radiation delivery from inability to account for differences in heart/thorax sizes between animals, this technique may also be less clinically relevant by delivering the radiation in only one or two fields [54]. Bazolava et al. have shown that the conformal systems, when compared to single-field irradiation without image-guidance, result in lower mean dose to tissues nearby the target region [54]. In a simulation of treatment plans for a mouse model bearing a lung tumor, they also showed that the mean dose to heart and the contralateral lung were lower when using a conformal, imagine-guided system to deliver radiation to the tumor, compared to single-field, non-image guided irradiation [54,62]. Other groups have also shown similar benefits of using image-guided, conformal radiation systems to target small areas like the heart [51,53,60,63].

Clinically, patients receiving thoracic RT often receive radiation to only part of the heart, instead of a fairly uniform radiation dose to the whole heart. While preclinical studies using whole heart irradiation have advanced our knowledge of RIHD and contributed to the mechanistic understanding of normal tissue radiation injury, whole heart radiation might not completely represent the clinical pathophysiology spectrum of RIHD [64,65,66,67]. Lee et al. showed that partial heart irradiation of ⅓ of the mouse left ventricle causes left ventricular dilation and increased fibrosis in the myocardium and pericardium, whereas the same phenotype is not observed with whole heart irradiation [66]. Lee et al. showed that partial heart irradiation of ⅓ of the mouse left ventricle causes left ventricular dilation and increased fibrosis in the myocardium and pericardium [67], whereas the same phenotype is not observed with whole heart irradiation [68]. The minimal fibrosis observed with the whole heart RT may be attributed to the rapid progression of myocardial necrosis and heart failure post-RT. The histological features of myocardial fibrosis and pericarditis seen in the partial-heart irradiation model seemed to mimic the changes observed in humans [67,69]. Moreover, numerous studies have shown relationships between the severity or specific pathophysiology of RIHD and the substructures of the heart receiving high doses of radiation [17,25,64,70,71,72]. While in previous clinical studies, mean heart dose was related to the likelihood of RIHD [12], and the risk of major coronary events in breast cancer patients increased linearly by approximately 4–16% for each 1 Gy in mean heart dose received [6,9,10], other studies have shown that dose to the coronary arteries may also be an indicator of risk of developing coronary artery stenosis, an important aspect of RIHD [73]. Thus, there is a need for irradiation of small segments of the heart in pre-clinical models, which may include key sections of coronary arteries, to accurately predict risk factors and successfully develop interventions in RIHD [64,74,75,76,77,78]. Partial heart irradiation in small animals may also be used to elucidate ways in which high-dose radiation treatment of segments of the heart can decrease ventricular tachycardia events in patients [79].

Partial heart irradiation in small animal models presents a myriad of potential technical obstacles that must be considered. For example, even with the advanced and widely available imaging modalities in clinics, researchers have found it difficult to delineate subregions of the heart based upon CT, especially non-contrast CT, which is the most widely used imaging modality for treatment planning [64]. The small size of experimental animal hearts makes precise identification of cardiac substructures in CT scans even more difficult than in the clinic. Moreover, the heart’s motion during respiratory and cardiac cycles as well as the complex anatomy and variability of the coronary vessels, adds to the challenges of standardizing subregion borders [11,64]. Newer imaging techniques like magnetic resonance imaging (MRI) might help overcome some of these challenges [80], but its use in RT treatment planning is limited clinically, and nearly absent in preclinical studies [64]. Furthermore, deep breath holding techniques or respiratory gating have been used clinically to decrease radiation dose to the heart [29,81], and similar techniques could potentially be useful for motion management in animals during precise heart and lung irradiation. However, since small animals will not hold their breath voluntarily and free-breathing gated treatment requires complex oversight during treatment administration, there are numerous technical challenges in using breath hold and other gating technique in preclinical studies. Overall, the cost, availability of advanced imaging modalities and software, and technical challenges in animal positioning, are some major limitations in 3D dose-volume mapping in small animals. After RT delivery, there are many methods available to obtain anatomic and physiologic information regarding cardiac function in preclinical studies. These include non-invasive methods such as echocardiogram, MRI, micro-CT, single-photon emission computerized tomography (SPECT), and positron emission tomography (PET) [67]. While imaging post-RT provides meaningful data in understanding how RT affects heart function, improved techniques to image while administering RT are greatly needed to improve the field of precise cardiac RT delivery.

### 2.2. Inconsistencies in Radiation Dose Delivery

Small animal radiation delivery to the whole heart or regions of the heart and/or lung, requires careful planning of dose distribution [82,83]. However, no standards have been followed for the performance and reporting of radiation dosimetry in small animal studies, which has made it difficult to reproduce previously published results [84]. In 2011, members of the Centers for Medical Countermeasures against Radiation (CMCR) outlined specific issues related to small animal irradiator calibration and dosimetry, in order to highlight the importance of “accurate and reproducible dosimetry” in preclinical radiobiology studies [85]. Gafchromic film dosimetry can be a valuable tool for characterization of small kV radiation beams [86]. However, even if the incident radiation field on a given animal is accurately assessed and uniform, the true delivered dose to the target volume will vary based on the incident radiation energy, the atomic number of the radiation source, and density of the tissue (e.g., lung, bone, soft-tissue) [85]. This effect can be difficult to measure and could potentially be overlooked depending on the geometry of the radiation field and the study goals. However, given the close interaction between the heart and lungs in the pathophysiology of radiation-induced toxicities (see also Section 2.3), and major differences in their tissue densities, these factors might be worth accounting for in preclinical studies that are aimed at isolating the effects within individual organs. For experiments where the exact absorbed dose is important to assess, researchers have developed microdosimetry techniques that allow calculation of non-homogeneous dose-distribution [87,88,89]. Additionally, in an effort to account for tissue-density based factors, groups have used Monte Carlo and image-based models to calculate absorbed dose [90,91,92].

Respiratory motion also adds complexity to the accurate delivery of thoracic RT. Studies have reported in mice a degree of motion in the order of 5 mm, and likely to be greater in rats [45,93]. The Verhaegen group performed a quantitative analysis of the impact of respiratory motion on a mouse lung tumor irradiation using a four-dimensional digital mouse whole body phantom. They reported respiratory motion resulted in an overestimation of a mean tumor dose of up to 11%, depending upon the placement of the tumor [94]. Similar errors in dose delivery may be expected in studies aiming to irradiate precise areas of normal tissues. Considering the heart in the context of respiration motion, radiation dose to the portions of the heart could either be overestimated or underestimated, as the heart moves in and out of the radiation field (see also Section 2.1). Therefore, respiratory motion is an important factor in considering treatment plans and improving clinical models. Efforts are underway to implement motion management or respiration gating in small animal RT [95,96], and temporary abdominal or chest compression may help to limit breathing motion in some small animals [97].

### 2.3. Cardiopulmonary Tissue Toxicity from RT

In addition to the heart, the lungs are also at risk of developing short- and long-term injuries after exposure to ionizing radiation [98,99,100]. In the pathogenesis of radiation-induced lung injury, several phases are recognized [100]. An early inflammatory phase is followed by the gradual development of radiation fibrosis and loss of lung function. Interestingly, studies in small animal models to determine mechanisms of radiation-induced lung injury face challenges similar to the ones described here for the heart [45]. The group of Coppes et al. have studied the interaction between lung and heart radiation injury by using proton beams to precisely irradiate the rat heart, alone or in combination with various volumes of the lung. The tolerance dose for loss of lung function was dependent upon the concomitant irradiation of the heart [101,102,103]. Conversely, manifestations of RIHD were more severe when both heart and lungs were exposed [66], and echocardiography after whole thorax or leg-out partial body irradiation revealed changes, including evidence of right-sided heart dysfunction, during periods when radiation pneumonitis was occurring [104,105]. Human studies have also suggested that both heart and lung doses can influence the development of cardiac and/or lung toxicities [14,106,107,108]. Thus, given the growing evidence that suggests that RIHD is linked to radiation-induced lung disease and/or lung doses received, it is important for researchers interested in understanding radiation injury in either organ alone to be aware of and control the dose volume to both the heart and lungs [65].

## 3. Models to Study Cardiac Radiation Toxicities: Animal Species, Strain, and Genotype

### 3.1. Animal Species Used in Preclinical Studies of RIHD

Historically, a number of pre-characterized mouse and rat models of cardiovascular disease have become available and used on a wide scale. The low maintenance and housing costs, gestational time and lifespan, and suitability for genetic selection and transgenic strain production, make rodents excellent models for proof-of-concept studies (Table 1). While rodent models are practical and provide us with critical insight into mechanisms by which radiation may injure the cardiovascular system, they do have some disadvantages. They are phylogenetically distant from humans, may have different physiologies and pathophysiologies, and can respond differently to pharmaceutical therapies [109,110,111,112]. However, many features of RIHD can be modeled in rodents, and much has been learned regarding the proteins and pathways involved in radiation-induced cardiotoxicity from these models [5,38]. Some of the limitations in rodents can be overcome by using rabbit models. Rabbits are more physiologically similar to humans from a cardiovascular standpoint (e.g., ion channel and Ca^2+^ transporter function), and are medium-sized animals, serving as a practical alternative to larger-sized animals [113]. Additionally, there are a myriad of transgenic rabbit models of cardiovascular disease, as well as several commercially available rabbit-specific antibodies, thereby making rabbit studies relatively feasible [113]. New Zealand White rabbits were first used by Fajardo and Stewart in 1968 to study RIHD [40]. The researchers concluded that the cardiac lesions developed in rabbit hearts after single dose x-ray exposure resembled the lesions seen in humans [40]. Rabbits have since only been used by a few researchers to study RIHD [41,114,115,116,117,118], although this species is commonly used to study cardiovascular disease from other causes [119,120,121,122,123,124,125]. There might be unique benefits to studying electrophysiological changes caused by radiation in rabbit models over rat models, as the rabbits’ Ca2+ transport, action potential duration, and main ionic currents underlying repolarization are similar to humans [113,126,127].

Despite the similarities in cardiovascular physiology between the rabbit and human, a major disadvantage in using both rabbit and rodent models include difference in the physical dimensions of the hearts, heart rates, and body weights when compared to humans. This might affect studies looking at arrhythmias [113], an effect of cardiac radiation exposure that has received renewed interest due to the use of radiation to treat ventricular tachycardia [79]. Additionally, certain molecular mechanisms of drug responses in large animals such as dogs and non-human primates are more similar to humans when compared to rodents, and thus may be better-suited for drug screening and toxicities [128,129]. Even among the larger animals, different species offer differing benefits. For example, models need to be selected carefully to address radiation-induced coronary alterations, an important component of human RIHD that needs additional study [6,122,123,124,125,126,127]. The coronary circulation of pigs is similar to young human hearts (e.g., no anastomoses between branches of the vasculature), while the coronary circulation in dogs is more similar to older human hearts with ischemic heart disease (highly collateralized circulation) [129,130,131]. These differences between species are important to keep in mind [6,132,133,134,135,136,137]. Few groups have used canine models for understanding the physiology of RIHD and for non-invasive imaging following local heart irradiation [138,139,140,141,142]. However, due to cultural values, unfavorable public opinion, more complex approval processes, and tight regulations in some countries, the use of canine models have decreased over time [143,144,145]. Other large animals such as non-human primates and pigs have been used by a few groups to study RIHD [146,147,148], but these are uncommon given the high cost and low throughput nature of large animal models. 

### 3.2. Influence of Strain on Manifestations of RIHD

In an effort to understand heritable genetic traits that could modify cardiac radiation sensitivity, our group utilized Salt-Sensitive (SS) and Brown Norway (BN) strain consomic rats. The inbred SS rat strain is more sensitive to RIHD than the inbred BN rat strain [49], as well as consomic SS.BN3 rats (genetically identical to SS rats except for chromosome 3, which is substituted from the BN rats) [49]. These results demonstrated that the BN rat chromosome 3 contains one or more genetic variations that play a protective role in the development of RIHD. These studies not only highlight the importance of heritable factors in determining the sensitivity radiation-induced cardiotoxicity, but also highlight the importance of selecting the proper rat strain for a particular research question [49]. Since studies in this field of research have made use of different mouse and rat strains to study the biological effects of cardiac irradiation [39,149,150], care must be taken when comparing outcomes from individual studies using difference strains.

### 3.3. Use of Genetically Modified Animals to Study Biological Mechanisms of RIHD

Genetically engineered mouse models allow researchers to mechanistically examine the effects of genetic changes on radiation damage in malignant and healthy tissue [151]. For example, researchers have used ApoE-deficient mice that are prone to atherosclerosis to study changes in inflammation and thrombosis following radiation delivery to the heart, aortic arch, and carotid arteries [152,153,154,155]. Lee et al. used mice with an endothelial cell-specific knock-out of p53 and p21 to understand the role of endothelial cells and the p53 pathway in radiation-induced heart damage [68]. The researchers used Cre-loxP technology to not only develop the p53-deficient mouse model, but also other mouse models that are more prone to radiation-induced normal tissue injury (i.e., faster onset, more prominent phenotype, etc.) [43,151] and models that are useful for studying cardiac damage following partial heart irradiation [67]. These genetically engineered mouse models are crucial for elucidating the role of specific signaling pathways and changes in the tissue microenvironment on RIHD, and the use of such models is expected to increase dramatically over time [151]. 

## 4. Preclinical Models to Study Cardiac Radiation Toxicities: Animal Age, Size, Dose/Fractionation, and Sex

As discussed above, attention must be paid to the choice of animal species, strain, and genotype when studying radiation-induced cardiotoxicity. However, studies show that additional variations in parameters such as animal age, size, and sex may influence outcome. These variables need to be accurately reported and considered in statistical analyses of results, as they may cause inconsistencies in findings between experimental groups within a research project and between separate studies. Radiation exposure at a young age has long been known to lead to more severe long-term normal tissue radiation injury compared to radiation exposure in adults [156]. Mulrooney et al. reported health outcomes in a cohort of just over 14,000 survivors of childhood and adolescent cancer and found that this population was at substantial risk for cardiovascular disease, with a two- to six-fold increased relative risk for coronary events in patients who had received >15 Gy to the heart compared to non-irradiated survivors [157]. Moreover, the relative risk of cardiovascular events was reported up to 60-fold higher in childhood cancer survivors that received a higher cardiac mean dose of >30 Gy compared to patients who received either no RT or a dose to the heart of 0.1 Gy or less [158]. While some studies report exposure at an older age may also be a risk factor for more severe normal tissue toxicity [159,160], a rodent study reported increased mortality rates and incidence of RT pneumonitis compared to geriatric rats after 13 Gy partial body irradiation [161]. Therefore, in preclinical research it is important to treat animals at an age that is most appropriate for the clinical scenario that is under investigation, which may also be dependent upon the sex and strain of the animal. However, many preclinical studies use young or young adult animals, due in large part to reduce the costs of the research.

A small number of clinical studies has addressed potential differences in the sensitivity to develop normal tissue radiation injury between male and female patients, including in the cardiopulmonary system [162,163,164,165]. Interestingly, as also described for preclinical animal models, an increased pulmonary radiation toxicity in women compared to men may be at least in part related to a smaller total lung volume in women and therefore a relatively larger percent volume of the female lung exposed to radiation [166,167]. The vast majority of preclinical studies of RIHD have used either male or female animals, and only few studies have included both sexes (examples include [49,168]). Because of the observations of an influence of sex on normal tissue radiation injury in human subjects, and with the increased call for studying both sexes in preclinical animal models, future studies should make direct comparisons between male and female animals in radiation-induced cardiopulmonary injury.

A number of different dose and fractionation regimens have been utilized in preclinical studies of RIHD. These range from single, large fractions of cardiac, to more clinically relevant fractionated regimens. However, due to the time and cost required for image-guided radiation therapy and potential anesthesia requirements of daily fractionated treatments, it may be difficult to model multi-week treatments using small fractions similar to commonly used clinical regimens (i.e., 1.8–2.7 Gy daily). Thus, a number of studies utilize larger daily fractions for more limited time periods, such as cardiac exposures to 9 Gy for five daily treatments [49,169].

## 5. Studying Cardiac Toxicity from Combined Cancer Therapies

Many cancer patients who receive RT as part of their treatment plan often also undergo surgery, chemotherapy, hormonal therapy, and/or immunotherapy. Numerous chemotherapy agents commonly used to treat thoracic cancers (e.g., doxorubicin) have been shown to cause cardiac toxicities on their own [170]. Whole heart x-ray exposure and Adriamycin (doxorubicin) have synergistic effects on heart toxicity in a New Zealand White rabbit model [171]. Therefore, additional cardioprotective measures might need to be taken prior to treatment or after therapy with radiation and anthracyclines. The cardiac effects of the combination of radiation with other anti-cancer agents are less commonly studied. For instance, while whole heart x-rays in combination with a tyrosine kinase inhibitor in rats may have more severe effects on cardiac mitochondrial structure than each of the treatments alone, long-term effects on cardiac function with these therapies are not yet known [169]. As more and more patients receive multiple and more diverse personalized therapies, further studies need to be conducted to study how multiple therapies interact to cause short- and long-term cardiovascular effects. In studying such combined therapies in animal models, one has to be careful in determining clinically relevant treatment doses and optimizing the order of the treatments and the time between the therapies.

### 5.1. Influence of Anesthesia on the Study of Combined Cancer Therapies

Experimental animals are often anesthetized prior to targeted radiation of the heart. However, the myocardial depressant effects of many anesthetics could potentially increase the severity of cardiotoxicity from chemotherapy, and possibly even radiation [172]. Additionally, anesthesia has been shown to reduce proliferation rates of natural killer cells and T-cells in rats and humans, which could potentially alter the effects of combining radiation and immunotherapy [173,174]. Thus, appropriate controls are necessary for understanding the effects of experimental conditions such as anesthesia.

### 5.2. Radiation Therapy and the Immune Response

The immune system has crucial housekeeping functions in the heart [175]. In some circumstances, such as after an infection or myocardial infarction, the immune system mediates healing and removal of dead tissue [175]. However, immune responses can also cause adverse tissue remodeling and irreversible damage [175,176]. For example, inflammation leads to induction of programmed death ligand 1 (PD-L1) in cardiac endothelial cells, which seems to protect the myocardium from cytotoxic T-lymphocyte-mediated cardiac injury [177]. Programmed death protein 1 (PD-1)-deficient mice develop autoimmune-mediated cardiomyopathy, but the development of cardiac disease is dependent upon the genetic background of the mouse model [177,178]. Thus, careful selection of animal strain and genetic background is crucial for accurate modeling of immune responses. These known roles of the PD-L1 pathway in adverse cardiac remodeling are also of significance in immune checkpoint inhibitor therapy. Ionizing radiation activates the anticancer immune response by exposing tumor-specific antigens and increasing tumor immunogenicity [179]. Moreover, it leads to the release of damage-associated molecular signals (including ATP, reactive oxygen species, heat shock proteins, and short DNA/RNA) following radiation-induced cell damage. These triggers mediate inflammation and innate immune responses [177,180]. Therefore, the combination of radiation with immune checkpoint inhibitors has increased anti-cancer response rates in preclinical and clinical studies. However, this combined treatment could also potentially have an adverse impact on normal tissues [181,182,183,184,185].

Du et al. studied RIHD in conjunction with inhibition of PD-1. C57BL/6 mice were given image-guided whole heart irradiation concurrently with PD-1 blockade. Increased mortality and cardiac dysfunction (illustrated by reduced ejection fraction) were observed in mice receiving cardiac RT with PD-1 blockade versus mice that only received RT [185]. Myers and Lu treated C57BL/6 mice with whole thorax irradiation in combination with an anti-PD-1 antibody and also observed reduced survival and increased numbers of T-cells in lung and heart compared to radiation alone [186]. These studies have shown that radiation-induced cardiac toxicity can be altered by PD-1 through cytotoxic T lymphocytes and suggest that PD-1 blockade should be administered with purposeful cardiac RT planning to ensure both positive treatment outcome and patient safety.

Although inbred mouse strains such as C57BL/6 mice have been widely used in anti-tumor and immunology studies, they have highly variable immune responses, and do not accurately mimic the human immune system [187]. Sanmamed et al. reviewed the benefits and shortcomings of various murine models for studying immune checkpoint inhibitors. The murine models that currently are closest to mimicking the human immune system are humanized mouse models, which have the most promise in testing the antitumor effects in immunotherapy strategies [188]. Humanized mouse models are developed using genetic, tissue, or environmental engineering methods, and have many immunologic factors that resemble humans [189]. However, the utility of a given model for studying RIHD is dependent on the method and type of immune engraftment. For example, CB-17-*scid*, NOD-*scid*, and NOG mice are highly suitable for oncological studies but have a low tolerance to radiation [190]. In studying normal tissue radiation toxicities in these mouse models, radiation doses may have to be reduced to obtain the same normal tissue pathologies as in common wild-type mice. Additionally, human microbiota have been shown to play a crucial role in the efficacy of cancer therapies and development of the host immune system [191,192,193,194]. While designing preclinical studies for combination therapies, particularly ones that require human immune responses, consideration of the immune population within humanized mice, differences in responses between inbred mice and humans, microbiota differences in animal models, and normal tissue radiation sensitivity should be considered.

### 5.3. Influence of Environmental Factors on Radiation Therapy

In addition to chemotherapy influencing RT responses and possible potentiation of side effects, a number lifestyle factors and environmental exposures may also alter RT side effects, yet preclinical studies to investigate these effects are thus far limited. For example, the effects of cigarette smoking during chemotherapy and/or RT revealed increased symptom burden compared to nonsmokers, including weight loss, skin problems, and nausea, in patients being treated for a number of different types of cancer [195]. In addition, studies have suggested that head and neck cancer patients who smoke during RT have lower rates of response and survival compared to patients that do not smoke [196]. The effects of smoking on cardiac RT sensitivity has not been well-studied, with little to no pre-clinical data reported.

Another environmental factor that may impact RT sensitivity is exercise, and exercise-oncology is an emerging area of interest as cancer therapy becomes more personalized. Again, clinical and preclinical studies to study this interaction are severely limited. Clinically, cardiorespiratory fitness was assessed following thoracic RT in breast or lung cancer patients that received significant heart exposure (>10% heart volume receiving 5 Gy). Patients displayed a dose-dependent relation between cardiac dose received and impairment in peak oxygen consumption, a marker of impaired cardiovascular reserve [197]. Exercise has also been reported to change redox signaling in cancer, as well as drive changes in immune system response, metabolism, and inflammation [198]. Because of the high level of redox signaling in the mitochondria and heart, a direct study to determine whether exercise influences cardiac sensitivity to RT would be valuable in the field regarding during cancer treatment as well as patient quality of life post-RT. Finally, diet has been implicated in affecting many diseases including heart disease and cancer. A preclinical study revealed fasting reduced intestinal radiotoxicity using C57BL/6J mice, assessed by fasting improved intestinal stem cell regeneration and improved survival to lethal doses of abdominal RT [199]. Musa and Shabeeb reported natural products may have a potential for protection against RIHD, reviewing products such as hesperidin, curcumin, melatonin, zingerone, and Shen-Mai San (SMS), where SMS is examined in a clinical trial for cancer patients receiving chemotherapy or RT (NCT01580358) [200]. Overall, there are currently limited published preclinical as well as clinical studies that investigate whether lifestyle factors affect incidence of RIHD.

## 6. Conclusions

Advances in the delivery of thoracic radiation therapy in preclinical models have translated into improved outcomes for cancer patients receiving RT. In return, advances in the clinic including image-guided irradiation have been implemented in research laboratories to further drive discoveries in the fields of cancer radiation therapy and normal tissue radiation toxicity. With current advancements in imaging, radiation delivery, and radiation dosimetry in preclinical research, we now have an opportunity to build our understanding of the normal tissue radiation response by improving the setup and dose distribution to better mimic clinical therapy. However, in each pre-clinical study this needs to be done with careful radiation dosimetry and reporting of the experimental setup to promote reproducibility. Lastly, understanding the differences and limitations of thoracic RT in preclinical models across research labs will aid in the proper interpretation of results and elucidation of pathways and mechanisms of radiation-induced cardiopulmonary damage.

## Figures and Tables

**Table 1:** Comparison of the main characteristics, advantages, and disadvantages of different animal species used in the study of RIHD. Items are ranked from highly optimal (++++) to less optimal (+).

Animal Model	Cost (Maintenance and Housing)	Gestational Time	Ease of Genetic Manipulation	Similarity of Cardiovascular System to Humans	Major Advantages	Major Disadvantages
Rodents	++++	++++	++++	+	Widely used, many genetically engineered and validated strains, can be humanized (immune system), useful for elucidating specific mechanisms of action.	Phylogenetically distant from humans, may respond differently to pharmaceutical therapies, have some differences physiology/pathophysiology from humans (e.g., rapid heart rate).
Rabbits	+++	+++	+++	++	Cardiac Ca^2+^ transporter function and ion channels are similar to humans, larger heart size makes more amenable to surgical- and catheter- based interventions, used in prior RIHD studies and commonly used in other cardiovascular studies.	Different physical dimensions of the heart, heart rates, and body weights when compared to humans (similar to rodents).
Canines	++	++	++	+++	More similar molecular mechanisms to humans, more suited for drug screening and toxicities, coronary circulation is similar to older human hearts with ischemic heart disease (high collateral circulation).	Unfavorable public opinion, complex approval process, high regulation in some countries.
Pigs	++	++	++	+++	More similar molecular mechanisms to humans, more suited for drug screening and toxicities, coronary circulation is similar to younger human hearts, used heavily in cardiovascular research.	Low throughput, long-duration of studies, cost.
Non-Human Primates	+	+	+	++++	Most similar to humans phylogenetically, anatomically, and physiologically.	Low throughput, long-duration of studies, ethical considerations, cost, more difficult and dangerous to work with.
